# Childcare Barriers and Appointment Nonadherence Among Women in a Safety-Net Health System

**DOI:** 10.1001/jamanetworkopen.2025.4715

**Published:** 2025-04-10

**Authors:** Anisha P. Ganguly, Robert Martin, Erin Barnett, Jillian Smartt, Michael Harms, Kimberly A. Kho, Michael E. Bowen, Bijal A. Balasubramanian, Kavita P. Bhavan

**Affiliations:** 1 Division of General Medicine and Clinical Epidemiology, Department of Internal Medicine, University of North Carolina at Chapel Hill; 2 Division of Maternal-Fetal Medicine, Department of Obstetrics and Gynecology, University of Texas Southwestern Medical Center, Dallas; 3 Center of Innovation and Value at Parkland, Parkland Health, Dallas, Texas; 4 Division of Gynecology, Department of Obstetrics and Gynecology, University of Texas Southwestern Medical Center, Dallas; 5 Division of General Internal Medicine, Department of Internal Medicine, University of Texas Southwestern Medical Center, Dallas; 6 O’Donnell School of Public Health, University of Texas Southwestern Medical Center, Dallas; 7 UTHealth Houston School of Public Health, Houston, Texas; 8 Division of Infectious Diseases, Department of Internal Medicine, University of Texas Southwestern Medical Center, Dallas

## Abstract

**Importance:**

Health-related social needs (HRSNs) contribute to appointment nonadherence. Childcare needs are an underrecognized HRSN that particularly affect women.

**Objective:**

To determine whether self-reported childcare barriers were associated with appointment nonadherence among women.

**Design, Setting, and Participants:**

This cross-sectional study used linked survey and electronic health record (EHR) data. Survey data were collected from November 2023 to May 2024 and EHR data were extracted for the 1 year prior to the survey date for each participant. Data were analyzed between June and September 2024. This single-center study was conducted in a safety-net health system in Dallas County, Texas. EHR data included ambulatory care encounters. Eligible participants included women aged 18 years or older referred to gynecology for abnormal cervical cancer screening who reported having childcare responsibilities.

**Exposure:**

Self-reported childcare barriers to appointments in the past year.

**Main Outcomes and Measures:**

Regression analysis was used to evaluate the primary outcome of appointment nonadherence rate during the year prior to survey date.

**Results:**

Of 1264 women eligible for the survey, 836 were successfully reached by telephone, of whom 671 women (53.1% of eligible sample) were included in the sample. Among the 671 initial respondents, 486 women reported having childcare responsibilities (72.4%). The mean (SD) age of the study sample was 34.8 (8.6) years, 405 (83.3%) identified as Hispanic, and 349 (71.8%) preferred Spanish. Additionally, 105 women reported childcare barriers to attending appointments in the past year (21.6%). Women with self-reported childcare barriers were mean 2.9 years younger and more likely to receive Medicaid (38 [36.2%]) than women without childcare barriers (89 [23.4%]). Women with self-reported childcare barriers were more likely to care for an increased number of children and younger children than women without. The mean (SD) appointment nonadherence rate was 25.1% (21.8%) among women with self-reported childcare barriers compared with 15.2% (22.1%) among women without. The adjusted difference in appointment nonadherence was 8.8 (95% CI, 3.6-14.0) percentage points (*P* = .001) higher among women with self-reported childcare barriers.

**Conclusions and Relevance:**

In this cross-sectional study of women in a safety-net health system, self-reported childcare barriers were associated with increased appointment nonadherence. Younger women with younger children were most likely to report childcare barriers to appointments. These findings highlight the importance of childcare needs as a HRSN that warrants screening and intervention.

## Key Points

**Question:**

Are childcare barriers associated with nonadherence to health care appointments?

**Findings:**

In this cross-sectional analysis of 486 women with childcare responsibilities in a safety-net patient population, 21.6% reported experiencing childcare barriers to appointments. Self-reported childcare barriers were significantly associated with an 8.8 percentage point increase in appointment nonadherence, and younger women with more children and younger children were more likely to report childcare barriers to health care appointments.

**Meaning:**

These findings suggest that childcare needs are a salient health-related social need that merits screening and interventions to promote appointment adherence for women.

## Teaser

This cross-sectional study assesses whether self-reported childcare barriers are associated with appointment nonadherence among women at a safety-net health system in Texas.

## Introduction

Health-related social needs (HRSNs) are immediate social and economic needs that affect the health of individuals. HRSNs are a downstream result of social drivers of health, the broader conditions that define health and well-being.^1^ HRSNs that limit access to medical care, such as insurance coverage, transportation, and work leave, are strongly associated with missed and deferred health care appointments (appointment nonadherence).^2,3,4^ HRSNs notably drive appointment nonadherence among patients with existing social vulnerability, including low income and racial or ethnic minority populations.^2,5,6^ Appointment nonadherence has been associated with worse clinical outcomes in a variety of clinical settings, including chronic disease management,^7,8^ cancer screening and treatment,^9,10^ and need for acute care management.^9^ Importantly, HRSNs that drive appointment nonadherence ultimately contribute to downstream differences in health outcomes, resulting in gender, racial and ethnic, and socioeconomic health disparities.^11,12^

Childcare needs are an underrecognized HRSN that can pose a barrier to appointments.^13^ Caregiving and childrearing responsibilities disproportionately fall to women^14,15^ and have important implications for gender equity in health care.^16^ The 2017 KFF Women’s Health Survey revealed that along with work leave and transportation, childcare barriers are a prevailing reason for missed or delayed medical care among women respondents.^17^ In our safety-net health system, we similarly found that childcare barriers were the most common reason for appointment nonadherence, and respondents rated access to childcare services as more difficult than access to health care services.^18^ Women from low income and minoritized racial or ethnic backgrounds are more likely to defer medical care due to competing responsibilities like childcare needs and experience difficulty accessing childcare services.^17,19^

Despite recent increasing attention to the role of caregiving as a social drivers of health, particularly among women,^16,20^ focused public health research about childcare barriers to health care remains lacking. In this study, we sought to further explore the association between childcare barriers and appointment nonadherence to examine childcare needs as an HRSN. We sought to provide a more precise assessment between childcare barriers and health care use, namely appointment nonadherence, measured with electronic health record (EHR) data. We hypothesized that women who reported experiencing childcare barriers to appointments would have a higher rate of appointment nonadherence compared with women who did not report childcare barriers.

## Methods

### Study Design and Setting

This cross-sectional study followed the Strengthening the Reporting of Observational Studies in Epidemiology (STROBE) reporting guideline. This study was approved by the University of Texas Southwestern institutional review board.

This is a cross-sectional study of baseline survey and EHR data collected from patients screened for eligibility for a pragmatic randomized clinical trial (RCT) of an intervention navigating women to a childcare resource to reduce appointment nonadherence in a cervical dysplasia clinic. The study setting for the RCT and this cross-sectional analysis was Parkland Health, a high-volume safety-net health system in Dallas County, Texas, serving one of the most highly uninsured urban populations in the country.^21,22^ All patients included in this study were referred for gynecological evaluation for abnormal cervical cancer screening per the clinical trial design. Women who were younger than 18 years, were currently incarcerated, and did not have children to care for were excluded.

Recruitment procedures for the clinical trial included a childcare screening survey took place from November 2023 to May 2024. The study team recruited patients over the phone and obtained verbal informed consent for participation in the screening survey.

### Data Source

Childcare needs and experiences were assessed using a de novo designed childcare screening survey instrument (eMethods in Supplement 1). The survey instrument included questions used in the 2017 KFF Women’s Health Survey^17^ and a similar needs assessment conducted in our health system in 2019,^18^ but was condensed to simplify data collection and focus the scope of the survey to childcare needs and barriers. The childcare screening survey included questions about childcare responsibilities, types of childcare relationships (eg, biological children, grandchildren, foster children), other sources of childcare support, and prior childcare methods used for accessing medical care. The survey was translated to Spanish using a validated translation service. The condensed survey instrument was administered to 10 patients in English and in Spanish for cognitive testing. The finalized survey instrument was administered to study participants over the telephone during the recruitment and data collection period. The survey was administered in English and Spanish when qualified Spanish-speaking research staff were available, and a telephone interpreter was used for non-English speaking patients where indicated. The primary exposure of interest was patient-reported childcare barriers experienced in the past year (eMethods in Supplement 1). Patients with only 1 child younger than 1 year at the time of survey were excluded for validity of the exposure variable, which asked patients to recall childcare barriers to appointments over the past year. Survey responses were compiled in REDCap.^23^

Demographic, clinical, and health care utilization variables were extracted from the EHR for patients enrolled in the survey. Race and ethnicity was included in this analysis to query disparate experiences of childcare barriers to appointment adherence. Race and ethnicity were obtained from the EHR, which is populated by patient self-report via patient portal or to administrative staff during patient registration. Race included options for American Indian or Alaska Native, Asian, Black or African American, Native Hawaiian or Pacific Islander, White or Caucasian, 2 or more races, and declined. Ethnicity included Hispanic and non-Hispanic. Race and ethnicity were combined in a composite variable that included Hispanic, non-Hispanic Black, non-Hispanic White, and other (including American Indian/Alaska Native, Asian, Other Pacific Islander, and unknown). Payer status was classified in the following categories: self-pay, Medicaid, county charity coverage, state charity coverage, and other. County charity coverage was comprised of patients receiving Dallas County-funded financial assistance, which includes coverage for ambulatory care, ED visits, hospitalizations, and medications, tiered to income levels up to 250% of the federal poverty limit (FPL).^24^ State charity coverage was comprised of uninsured patients receiving coverage for cervical cancer screening through the Texas Breast and Cervical Cancer Screening Program,^25^ a program funded by the National Breast and Cervical Cancer Early Detection Program,^26^ administered through Texas Medicaid but limited only to cancer screening services.

### Outcomes and Covariates

The primary outcome of interest was appointment nonadherence rate, which was defined as the composite of outpatient appointment EHR encounters coded as missed or patient-initiated cancellation divided by the number of scheduled appointments within the last year. Appointments cancelled by a clinician were excluded. Appointment types were limited to outpatient clinician appointments and were classified in the following appointment categories: primary care, women’s health, specialty care, procedures, imaging, and telehealth. Women’s health appointments included women’s health nurse practitioner (WHNP) and obstetrics and gynecology (OB/GYN) appointments. Telehealth appointments included medical and mental health appointments.

Covariates were selected a priori by the study team as potential confounders for the primary outcome of appointment nonadherence rate. Covariates included age, relationship status, preferred language, payer status, and comorbidities.

### Statistical Analysis

Baseline characteristics and childcare survey variables were represented with descriptive statistics. We compared patients with self-reported childcare barriers in the past year with those who did not report experiencing childcare barriers using χ^2^ and Kruskal-Wallis tests. Univariable and multivariable linear regression were performed to test the association between patient-reported childcare barriers and appointment nonadherence rate over the year prior, adjusting for age, relationship status, preferred language, payer status, and diagnosis of diabetes. Linearity assumptions were assessed by confirmation of homoscedasticity using the White test, normality of regression residuals, and lack of multicollinearity confirmed by variance inflation factors less than 2. All statistical tests were 2-sided and significance was defined at α < .05. All analyses were completed in Python version 3.11 (Python Software Foundation). Data were analyzed between June and September 2024.

## Results

### Patient Characteristics

Of 1264 women eligible for the survey, 836 were successfully reached by telephone, of whom 671 (53.1% of eligible sample) consented to and completed the survey (eFigure in Supplement 1). Among 671 women surveyed, 486 reported having childcare responsibilities (72.4%) and were included in the analysis. Within the study population of 486 respondents with childcare responsibilities, 105 reported experiencing childcare barriers to health care appointments within the past year (21.6%), and 381 did not report childcare barriers (78.4%) (Table 1). Overall, women with self-reported childcare barriers in the past year were more likely than women without childcare barriers to be younger in age (mean, 2.9 years younger; *P* = .008), receive Medicaid for coverage (38 [36.2%] vs 89 [23.4%]), and be pregnant within the year prior to survey (43 [41.0%] vs 100 [26.2%]) compared with women without childcare barriers. There were no significant differences in race or ethnicity or preferred language based on self-reported childcare barriers. There were no significant differences in diagnosis of diabetes, hypertension, or HIV. There were no differences in having a known primary care clinician (PCC) or number of scheduled PCC appointments in the past year.

**Table 1.** Baseline Characteristics of Women With and Without Self-Reported Childcare Barriers

Characteristic	Patients, No. (%)			*P* value
	Total (N = 486)	Women		
		Without childcare barriers (n = 381)	With childcare barriers (n = 105)	
Age, mean (SD), y	34.8 (8.6)	35.4 (8.9)	32.5 (6.9)	.008
Race and ethnicity				
Hispanic	405 (83.3)	321 (84.3)	84 (80.0)	.16
Non-Hispanic Black	59 (12.1)	42 (11.0)	17 (16.2)	
Non-Hispanic White	13 (2.7)	9 (2.4)	4 (3.8)	
Other^a^	9 (1.9)	9 (2.4)	0	
Preferred language				
English	132 (27.25)	95 (24.9)	37 (35.2)	.11
Spanish	349 (71.8)	282 (74.0)	67 (63.8)	
Other^b^	5 (1.0)	4 (1.0)	1 (1.0)	
Payer status				
County charity coverage	64 (13.2)	49 (12.9)	15 (14.3)	.045
Medicaid	127 (26.1)	89 (23.4)	38 (36.2)	
State charity coverage^c^	222 (45.7)	183 (48.0)	39 (37.1)	
Self-pay	73 (15.0)	60 (15.7)	13 (12.4)	
Marital status				
No partner or other	294 (60.5)	228 (59.8)	66 (62.9)	.58
Married or significant other	192 (39.5)	153 (40.2)	39 (37.1)	
Comorbidities				
Diabetes	20 (4.1)	12 (3.1)	8 (7.6)	.08
Hypertension	29 (6.0)	22 (5.8)	7 (6.7)	.91
HIV	9 (1.9)	5 (1.3)	4 (3.8)	.20
Known PCC				
Yes	180 (37.0)	138 (36.2)	42 (40.0)	.48
No	306 (63.0)	243 (63.8)	63 (60.0)	
PCC visits scheduled in past year, mean (SD)	1.5 (2.8)	1.4 (2.8)	1.6 (2.7)	.87
PCC appointments scheduled in past 3 y, mean (SD)	3.0 (6.6)	3.0 (6.8)	2.7 (5.4)	.99
Pregnancy status				
Current pregnancy	76 (15.6)	61 (16.0)	15 (14.3)	.67
Pregnancy in the past year	143 (29.4)	100 (26.2)	43 (41.0)	.003
Pregnancy in the past 5 y	63 (13.0)	43 (11.3)	20 (19.0)	.04

Abbreviation: PCC, primary care clinician.

^a^ Other race or ethnicity included American Indian and Alaska Native (n = 1), Asian (n = 3), Other Pacific Islander (n = 1), and unknown (n = 4).

^b^ Other preferred language included Arabic (n = 1), Tigrinya (n = 1), Yoruba (n = 1), and unknown (n = 2).

^c^ In the state of Texas, eligible uninsured women may receive state coverage for cancer screening under the Breast and Cervical Cancer Screening program, which is administered under Texas Medicaid. These patients are classified separately from Medicaid patients because they do not receive other Medicaid benefits.

### Characteristics of Childcare Needs and Experiences

Responses to the childcare screening survey are provided in Table 2. Women with self-reported childcare barriers were more likely to care for a greater number of children and for children younger in age compared with women without childcare barriers. Self-reported childcare barriers to health care appointments were most often attributed to childcare needs related to respondents’ own children; women who reported childcare responsibilities for grandchildren, nieces and nephews, and others were unlikely to report experiencing childcare barriers to appointments. Sources of caregiving support (spousal and partner support, parents of mothers, or family and community networks) were similar among both groups. Those with self-reported childcare barriers were more likely that those without childcare barriers to bring their children to appointments (30 [28.6%] vs 64 [16.8%]; *P* = .007) or schedule their appointments during school hours (55 [52.4%] vs 144 [37.8%]; *P* = .007). Overall, use of paid babysitting and daycare services were low across both groups, and both groups reported commonly using family support for childcare needs during health care appointments. Women with self-reported childcare barriers in the past year were much more likely to report an anticipated childcare need for their upcoming cervical dysplasia appointment compared with women who did not report childcare barriers in the past (36 [34.6%] vs 27 [7.1%]; *P* < .001).

**Table 2.** Characteristics of Childcare Needs Among Women With and Without Self-Reported Childcare Barriers

Characteristics of childcare needs	No. (%)			*P* value
	Total (N = 486)	Women		
		Without childcare barriers (n = 381)	With childcare barriers (n = 105)	
No. of children				
1	156 (32.1)	136 (35.7)	20 (19.0)	.02
2	154 (31.7)	114 (29.9)	40 (38.1)	
3	114 (23.5)	83 (21.8)	31 (29.5)	
4	37 (7.6)	30 (7.9)	7 (6.7)	
≥5	25 (5.1)	18 (4.7)	7 (6.7)	
Age of children, y^a^				
<1	154 (14.9)	108 (13.7)	46 (18.5)	.006
1-3	105 (10.2)	72 (9.2)	33 (13.3)	
3-5	104 (10.1)	76 (9.7)	28 (11.3)	
5-8	189 (18.3)	139 (17.7)	50 (20.2)	
8-11	173 (16.7)	132 (16.8)	41 (16.5)	
11-14	157 (15.2)	129 (16.4)	28 (11.3)	
14-18	152 (14.7)	130 (16.5)	22 (8.9)	
Childcare relationships				
Own children	454 (93.4)	350 (91.9)	104 (99.0)	.02
Nieces or nephews	21 (4.3)	18 (4.7)	3 (2.9)	.57
Grandchildren	32 (6.6)	31 (8.1)	1 (1.0)	.02
Other	15 (3.1)	11 (2.9)	4 (3.8)	.87
Other caregivers in the family or community				
Sole caregiver for children	96 (19.8)	72 (18.9)	24 (22.9)	.37
Spouse or partner of respondent	159 (32.7)	117 (30.7)	42 (40.0)	.08
Parents of respondent	100 (20.6)	80 (21.0)	20 (19.0)	.66
Older children	55 (11.3)	46 (12.1)	9 (8.6)	.41
Nonpartnered parents of child	21 (4.3)	16 (4.2)	5 (4.8)	>.99
Friends or neighbors	124 (25.5)	98 (25.7)	26 (24.8)	.84
Other	89 (18.3)	78 (20.5)	11 (10.5)	.02
Prior childcare methods for appointments				
Bringing children to appointments	94 (19.3)	64 (16.8)	30 (28.6)	.007
Scheduling during school hours	199 (40.9)	144 (37.8)	55 (52.4)	.007
Paid babysitter	41 (8.4)	28 (7.3)	13 (12.4)	.10
Paid daycare	11 (2.3)	7 (1.8)	4 (3.8)	.41
Family support	248 (51.0)	190 (49.9)	58 (55.2)	.33
Friends or neighbor support	33 (6.8)	23 (6.0)	10 (9.5)	.21
Other	23 (4.7)	20 (5.2)	3 (2.9)	.45
Anticipated childcare barrier for upcoming cervical dysplasia appointment^b^				
Yes	63 (13.1)	27 (7.1)	36 (34.6)	<.001
No	419 (86.9)	351 (92.9)	68 (65.4)	

^a^ There were 786 children among women without childcare barriers, and 248 children among women with childcare barriers.

^b^ For this question, 482 patients responded. Four patients in the total sample did not provide a response to this question.

### Appointment Nonadherence

Appointment outcomes are reported in Table 3. Women with self-reported childcare barriers had a higher number of scheduled appointments in the past year compared with women without childcare barriers (mean increase of 1.5 scheduled appointments; *P* = .02). The most common scheduled appointment type was women’s health appointments; women with self-reported childcare barriers had a mean of 1.0 more women’s health appointments than women without (*P* = .02). Among women with self-reported childcare barriers, the appointment nonadherence rate was 25.1% (21.8%), compared with 15.2% (22.1%) among women without childcare barriers, resulting in an unadjusted difference of 9.9% in appointment nonadherence. Among outpatient appointment types, the prevailing differences in nonadherence rate related to self-reported childcare barriers were in women’s health, primary care, and imaging appointments.

**Table 3.** Unadjusted Appointment Outcomes Among Women With and Without Self-Reported Childcare Barriers

Appointment outcomes	Women, mean (SD)			*P* value
	Total (N = 486)	Without childcare barriers (n = 381)	With childcare barriers (n = 105)	
**No. of appointments scheduled in past year**				
All appointment types	7.1 (6.2)	6.8 (6.1)	8.3 (6.5)	.02
Primary care appointments	0.67 (1.3)	0.67 (1.3)	0.68 (1.5)	.34
Women’s health appointments	4.4 (4.5)	4.2 (4.5)	5.2 (4.3)	.02
Specialty care appointments	0.25 (1.3)	0.24 (1.3)	0.28 (0.96)	.13
Procedure appointments	0.26 (1.1)	0.22 (0.95)	0.38 (1.7)	.82
Imaging appointments	0.58 (1.5)	0.58 (1.5)	0.59 (1.5)	.49
Telehealth appointments	0.89 (1.4)	0.81 (1.3)	1.2 (1.6)	.04
**Appointment nonadherence rate in past year, %**				
All appointment types	17.4 (22.4)	15.2 (22.1)	25.1 (21.8)	<.001
Primary care appointments	17.9 (34.8)	15.1 (33.2)	30.4 (39.7)	.01
Women’s health appointments	16.6 (25.3)	14.3 (24.7)	24.5 (25.7)	<.001
Specialty care appointments	26.2 (37.4)	23.5 (36.2)	31.8 (41.2)	.59
Procedure appointments	8.9 (26.1)	10.1 (28.7)	5.0 (14.0)	.97
Imaging appointments	13.2 (28.5)	9.6 (24.8)	28.5 (37.8)	.002
Telehealth appointments	16.6 (30.2)	16.3 (30.7)	17.6 (29.0)	.50

Multivariable linear regression was used to understand the association between self-reported childcare barriers in the past year and appointment nonadherence rate within the past year (Table 4). After adjusting for respondent age, relationship status, preferred language, payer status, and diagnosis of diabetes, the adjusted difference in appointment nonadherence rate among women with self-reported childcare barriers was 8.8 (95% CI, 3.6 to 14.0) percentage points (*P* = .001). Non-English preferred language was also significantly negatively associated with appointment nonadherence at an adjusted rate of −6.2 (95% CI, −11.1 to −1.2) percentage points (*P* = .01). Respondent age, relationship status, payer status, and diagnosis of diabetes were not associated with appointment nonadherence. A sensitivity analysis (eTable in Supplement 1) exploring the association between self-reported childcare barriers and no-show rate alone without patient-initiated cancellation rate showed a similar adjusted increase in no-show rate among women with self-reported childcare barriers (8.2 [95% CI, 3.4 to 13.0] percentage points; *P* = .001).

**Table 4.** Multivariable Linear Regression of Self-Reported Childcare Barriers and Appointment Nonadherence

Variable	Nonadherence rate, adjusted % (95% CI), percentage points	*P* value
Self-reported childcare barriers		
Yes	8.8 (3.6 to 14.0)	.001
No	[Reference]	NA
Age	−0.2 (−0.4 to 0.1)	.25
Relationship status		
Single	3.3 (−1.1 to7.7)	.14
Has partner	[Reference]	NA
Preferred language		
English	[Reference]	NA
Spanish/other	−6.2 (−11.1 to −1.2)	.01
Payer status		
State charity coverage	2.6 (−2.7 to 7.9)	.34
County charity coverage	−2.6 (−9.9 to 4.6)	.75
Other	0.5 (−7.4 to 6.4)	.86
Medicaid	[Reference]	NA
Diagnosis of diabetes		
Yes	−4.6 (−15.3 to 6.2)	.40
No	[Reference]	NA

Abbreviation: NA, not available.

## Discussion

In this cross-sectional study of linked survey and EHR data, we found that women with self-reported childcare barriers had an adjusted 8.8% higher rate of appointment nonadherence compared with women without childcare barriers. To our knowledge, this analysis is the first study to demonstrate an association between patient-reported childcare barriers and an objective assessment of appointment nonadherence from EHR data. Our study also provided important formative characterization of how childcare barriers manifest, who childcare barriers affect, and how women navigate childcare barriers to appointments. These study findings highlight the clinical impact experienced by women with childcare needs that pose a barrier to attending and completing health care appointments. The quantifiable difference in appointment adherence demonstrated in this analysis supports the assertion that, much like the barriers posed by transportation^27,28^ and work leave,^29,30^ childcare needs were a clinically significant HRSN.

We found that patient-reported childcare barriers were associated with an increased rate of missed and cancelled appointments, persistent after adjusting for age, payer status, and other covariates associated with appointment nonadherence.^31^ This finding suggests an association between patient-reported experience of childcare needs interfering with appointment attendance and objective EHR health care utilization data demonstrating missed and cancelled appointments. This association builds upon prior survey data and qualitative analyses demonstrating how childcare needs affect health care access,^17,32^ particularly as a barrier to appointment adherence.^33,34^ Prior research has demonstrated a difference of 5% to 10% for patients reporting 1 or more HRSNs and appointment nonadherence,^3,35^ similar to the difference of 8.8% noted in this analysis.

Notably, consistent with a prior childcare needs assessment in the Parkland Health system,^18^ we found that the prevailing missed and/or cancelled appointments among women reporting childcare barriers were women’s health and primary care appointments, suggesting that among health care needs, preventive care is most affected by childcare barriers in this population. This finding is supported by prior research that found a pattern among women of forgoing preventive care due to competing social needs.^33,36^ Such psychosocial stressors have been shown to particularly impact preventive care use among women of low income and racially or ethnically minoritized backgrounds,^37,38^ much like the patients included in this study sample. While preventive care may be deprioritized in the face of competing needs, HRSNs like childcare barriers may contribute to disparities in preventive care, including vaccinations, cancer screenings, and chronic disease management.^39,40^

This study also further contextualized childcare needs as a HRSN. This study showed that childcare barriers were more common among younger women with younger children and more children for whom to care. While there were women who reported childcare responsibilities for children other than their own biological children (eg, grandchildren), those childcare relationships were not found to pose a barrier to attending appointments. The survey showed important findings about how women navigate childcare barriers for scheduled health care appointments, namely reliance on school schedules to coordinate their own health care and engagement of family and social networks for childcare support. These findings were consistent with longstanding research demonstrating reliance on informal care and community caregiving for childcare needs among low income and minoritized mothers.^41,42,43^ Of note, more than 25% of women reporting childcare barriers to appointments reported bringing children to their own appointments due to lack of childcare, a concerning finding similarly demonstrated in the Veterans Health Administration.^34^

The findings of this study have potential implications for gender equity in health care. Amid the US health care system’s reckoning with screening for and addressing social drivers of health as part of health care delivery,^44,45^ childcare needs remain absent or peripheral in standardized assessments of HRSNs.^46,47^ A recent scoping review from our group found a limited number of publications exploring childcare needs as associated with the health of caregivers, and among those publications, more than half examined childcare alongside other HRSNs, not as a distinct, unique social driver of health.^13^

Recognizing that childcare responsibilities disproportionately fall to women, it was not surprising that the role of childcare barriers in health care remains underappreciated in the broader discussion of HRSN and social drivers of health. This analysis is an important step to advance public health understanding about access to childcare as a driver of inequitable outcomes among women and may have significance for ongoing research in gender disparities.^48,49,50,51^ Additionally, the impact of childcare barriers on health care access is an important contribution to ongoing national conversations on childcare policy.^52,53^ Current discourse on childcare policy reform emphasizes the impact of childcare availability and affordability on parental workforce engagement^54,55,56,57^; this study suggests that childcare policy may affect not only employment, but also health outcomes of mothers.

### Limitations

This study has limitations. This is a cross-sectional study that relied on linked survey and EHR data. The cross-sectional study design does not demonstrate causal effects between childcare barriers and appointment nonadherence, and other unaccounted factors in this analysis (eg, transportation barriers, financial strain beyond documented payer status) may contribute to the demonstrated differences. Similar to prior studies exploring childcare needs through survey methods, the variables collected through the childcare screening survey may be subject to recall bias. The study sample is derived from a single safety-net health system in Dallas County serving a predominantly low income, highly uninsured patient population, which may not generalize to other settings. Nonetheless, representation of this socially vulnerable population serves to amplify the capture of HRSNs, including childcare needs and highlight the challenges faced by similarly marginalized communities. Of note, the sample in this study included women with cervical dysplasia screened for eligibility in an RCT, which likely limits generalizability to the broader population of women, such as women seeking primary care. However, given prior research suggesting an association between social vulnerability, HRSNs, and cervical cancer risk and nonadherence to screening,^58,59,60^ this patient sample was a particularly high-risk population for HRSNs and well suited to explore undercharacterized social needs like childcare. Future research is needed to understand the prevalence of childcare barriers in other populations and further elucidate the mechanisms that childcare barriers play in access to health care.

## Conclusions

In this cross-sectional study of women in a safety-net health system, patient-reported childcare barriers were associated with increased nonadherence to scheduled health care appointments. Childcare barriers were more common among women who were younger, had younger children, and had more children. This study suggests that childcare needs may pose a clinically relevant barrier to women completing necessary health care appointments, which may affect health outcomes. Additional research is needed to screen for, understand, and overcome childcare barriers to support access and use of health care services among women.

## Notes

Supplement 1.
eMethods.
**eFigure.** Study Flow Diagram of Patient Recruitment and Enrollment in Childcare Screening Survey **eTable.** Sensitivity Analysis of Self-Reported Childcare Barriers and No-Show Rate

Supplement 2.
Data Sharing Statement
