# Supplementary material for: Childcare Barriers and Appointment Nonadherence Among Women in a Safety-Net Health System
Source: JAMA Netw Open. 2025 Apr 10;8(4):e254715. doi: 10.1001/jamanetworkopen.2025.4715 (PMC11986774; doi:10.1001/jamanetworkopen.2025.4715)
Supplement: Supplement 1. — eMethods. eFigure. Study Flow Diagram of Patient Recruitment and Enrollment in Childcare Screening Survey eTable. Sensitivity Analysis of Self-Reported Childcare Barriers and No-Show Rate [file jamanetwopen-e254715-s001.pdf]

Supplemental Online Content

Ganguly AP, Martin R, Barnett E, et al. Childcare barriers and appointment nonadherence among women in a safety-net health system. *JAMA Netw Open*. 2025;8(4):e254715. doi:10.1001/jamanetworkopen.2025.4715

eMethods.

eFigure. Study Flow Diagram of Patient Recruitment and Enrollment in Childcare Screening Survey

eTable. Sensitivity Analysis of Self-Reported Childcare Barriers and No-Show Rate

This supplemental material has been provided by the authors to give readers additional information about their work.

© 2025 Ganguly AP et al. *JAMA Network Open*.

eMethods. Childcare Screening Survey Instrument

1. Do you have children that you take care of?
    a. Yes
    b. No *(If no, thank them for their time and end the call).*
2. How many children do you take care of? *(Fill in the correct number)*
3. How old are each of the children that you take care of? *(Fill in the ages for each child reported in #2).*
4. Are the children that you care for (check all that apply):
    a. Your children
    b. Nieces/nephews
    c. Grandchildren
    d. Foster children
    e. Children of friends/neighbors
    f. Children that you are paid to take care of
    g. Other (please specify)
5. Who else in your family or community helps with childcare duties (check all that apply):
    a. Spouse/partner
    b. Parents
    c. Older children
    d. Parents of children
    e. Friends/neighbors
    f. Other (please specify)
6. (If responds yes to older children in #5) How old are your older children?
7. In the past year, have you missed or cancelled an appointment due to needing childcare?
    a. Yes
    b. No
8. In the past year have you missed or cancelled one of these types of appointments due to childcare?
    a. Primary care (wellness visit, vaccines, cancer screening, etc.)
    b. Specialty care (example: cardiology, diabetes, optometry)
    c. Women's health
    d. Procedure
    e. Other (please specify)
    f. None of the above
9. When you have a medical appointment, how do you get childcare for your children? (check all that apply)
    a. Bring child(ren) to visits
    b. Schedule appointments during school hours
    c. Pay for a babysitter
    d. Pay for a daycare center
    e. Family helps
    f. Neighbors/friends help
    g. Other (please specify)
    h. None

© 2025 Ganguly AP et al. *JAMA Network Open*.

10. What did you do for childcare during your recent cervical cancer screening visit (Pap test/Pap smear)?
    a. Brough child(ren) to the appointment
    b. Appointment was during school hours
    c. Paid for a babysitter
    d. Paid for a daycare center
    e. Family took care of the child(ren)
    f. Neighbor/friend took care of the child(ren)
    g. Don’t remember
    h. Other
11. Do you have a plan for childcare for your upcoming gynecology dysplasia appointment?
    a. Yes
    b. No
    c. Maybe
12. Do you need childcare for your upcoming gynecology dysplasia appointment?
    a. Yes
    b. No

© 2025 Ganguly AP et al. *JAMA Network Open*.

eFigure. Study Flow Diagram of Patient Recruitment and Enrollment in Childcare Screening Survey

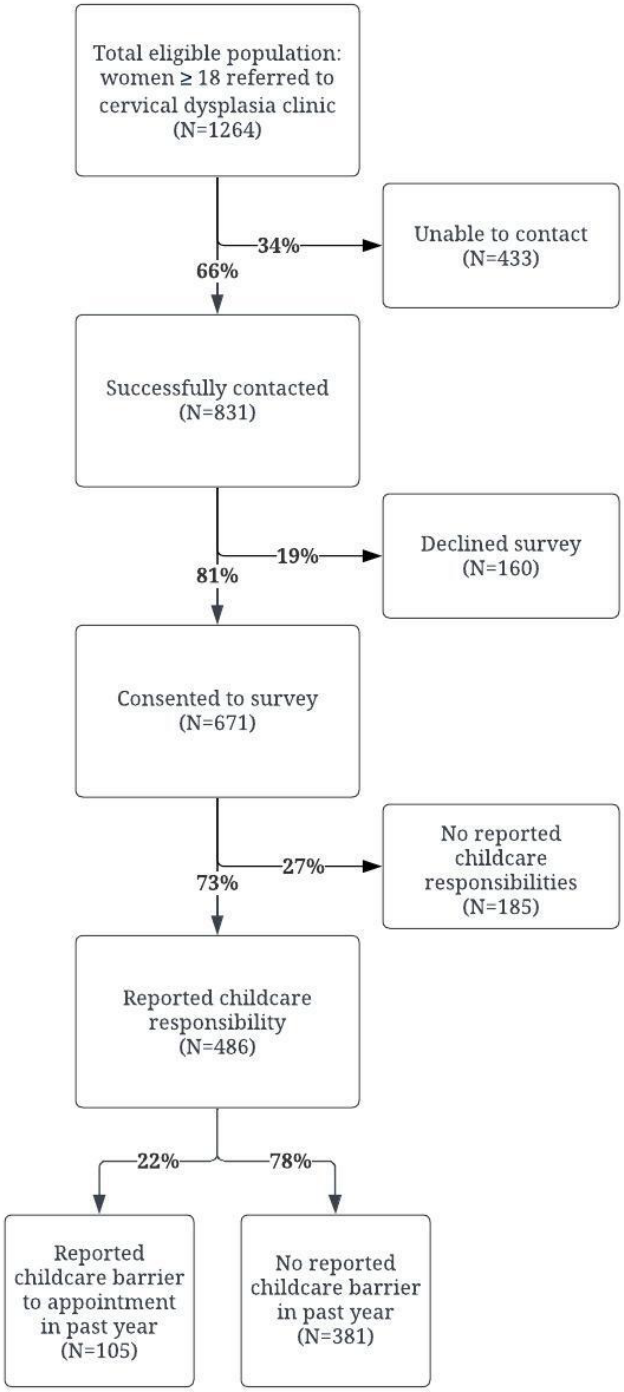

© 2025 Ganguly AP et al. *JAMA Network Open*.

eTable. Sensitivity Analysis of Self-Reported Childcare Barriers and No-Show Rate

**Variable**		**Adjusted % non-adherence rate (95% CI)**	
		**Primary outcome: composite of no-show + patient-initiated cancellation**	**No-show only**
**Self-reported childcare barriers**	Yes	8.8 (3.6 to14.0)	8.2 (3.4 to 13.0)
	No (*ref*)	--	--
**Age**		-0.2 (-0.4 to 0.1)	-0.1 (-0.4 to 0.1)
**Relationship Status**	Single	3.3 (-1.1 to7.7)	1.0 (-3.1 to 5.1)
	Has Partner (*ref*)	--	--
**Preferred Language**	English (*ref*)	--	--
	Spanish/other	-6.2 (-1.2 to -11.1)	-4.1 (-8.6 to 0.5)
**Payer status**	State Charity Coverage	2.6 (-2.7 to 7.9)	0.8 (-4.2 to 5.7)
	County Charity Coverage	-2.6 (-9.9 to 4.6)	-3.4 (-10.1 to 3.4)
	Other	0.5 (-7.4 to 6.4)	-1.0 (-7.4 to 5.4)
	Medicaid (*ref*)	--	--
**Diagnosis of diabetes**	Yes	-4.6 (-15.3 to 6.2)	-7.9 (-17.9 to 2.0)
	No (*ref*)	--	--

© 2025 Ganguly AP et al. *JAMA Network Open*.
