# Supplementary material for: Childcare Barriers and Appointment Nonadherence Among Women in a Safety-Net Health System
Source: JAMA Netw Open. 2025 Apr 10;8(4):e254715. doi: 10.1001/jamanetworkopen.2025.4715 (PMC11986774; doi:10.1001/jamanetworkopen.2025.4715)
Supplement: Supplement 2. — Data Sharing Statement [file jamanetwopen-e254715-s002.pdf]

Data Sharing Statement

Data
Data available: No

Additional Information
Explanation for why data not available: Data sharing of this dataset of individual patient survey data linked with EMR data is not permissible by the study health system's Office of Research Administration.
